# Supplementary material for: Massive antibody discovery used to probe structure–function relationships of the essential outer membrane protein LptD
Source: eLife. 2019 Jun 25;8:e46258. doi: 10.7554/eLife.46258 (PMC6592684; doi:10.7554/eLife.46258)
Supplement: Supplementary file 1. — Bacterial growth curves in Figure 3 and Figure 3—figure supplement 1 were analyzed by determining the doubling time (dt) during exponential growth phase and compared via the unpaired Student’s t test. The Bonferroni correction was applied to control for multiple comparisons. [file elife-46258-supp1.docx]

**Storek et al. Supplementary File 1**

**Table. Statistical analysis of the initial growth rates for conditional *lptD* deletion strains complemented with *lptD* loop mutants.**

|  |  |  | **complementing lptD vector^1^** | | | | | | | | | | | | | | |
| --- | --- | --- | --- | --- | --- | --- | --- | --- | --- | --- | --- | --- | --- | --- | --- | --- | --- |
| **strain** | **condition** | **calculation** | **VC** | **WT** | **ΔL1** | **ΔL2** | **ΔL3** | **ΔL4** | **ΔL5** | **ΔL6** | **ΔL7** | **ΔL8** | **ΔL9** | **ΔL10** | **ΔL11** | **ΔL12** | **ΔL13** |
| wild-type | no arabinose | dt (h)^2^ | nd | 1.22 | 1.84 | 5.51 | 2.09 | 1.73 | 1.45 | 1.40 | 1.79 | 1.58 | 1.53 | 5.84 | 1.55 | 1.62 | 1.55 |
|  |  | dt (SD)^3^ | nd | 0.01 | 0.04 | 0.90 | 0.03 | 0.04 | 0.08 | 0.02 | 0.18 | 0.07 | 0.04 | 0.30 | 0.11 | 0.05 | 0.07 |
|  |  | *t*-test^4^ |  |  | <0.0001 | 0.0012 | <0.0001 | <0.0001 | 0.0083 | 0.0002 | 0.006 | 0.0008 | 0.0002 | <0.0001 | 0.0062 | 0.0002 | 0.0013 |
|  |  | Significant^5^ |  |  | yes | yes | yes | yes | no | yes | no | yes | yes | yes | no | yes | yes |
| wild-type | 0.02% arabinose | dt (h) | 1.44 | 1.37 | 1.48 | 1.46 | 1.56 | 1.51 | 1.53 | 1.54 | 1.51 | 1.53 | 1.57 | 1.51 | 1.43 | 1.45 | 1.61 |
|  |  | dt (SD) | 0.09 | 0.06 | 0.04 | 0.03 | 0.04 | 0.09 | 0.10 | 0.06 | 0.09 | 0.08 | 0.06 | 0.07 | 0.02 | 0.05 | 0.07 |
|  |  | *t*-test | 0.3832 | - | 0.0843 | 0.1288 | 0.0153 | 0.0903 | 0.0815 | 0.0307 | 0.1 | 0.0698 | 0.0186 | 0.0828 | 0.2269 | 0.1705 | 0.014 |
|  |  | Significant | no |  | no | no | no | no | no | no | no | no | no | no | no | no | no |
| Δ*waaD* | no arabinose | dt (h) | nd | 2.58 | 1.74 | 1.96 | 2.80 | 2.16 | 2.48 | 2.01 | 2.42 | 1.88 | 2.28 | nd | 2.45 | 2.20 | 2.56 |
|  |  | dt (SD) | nd | 0.28 | 0.16 | 0.35 | 0.19 | 0.13 | 0.10 | 0.05 | 0.26 | 0.11 | 0.08 | nd | 0.08 | 0.12 | 0.06 |
|  |  | *t*-test |  |  | 0.0107 | 0.0779 | 0.3118 | 0.0797 | 0.6166 | 0.0259 | 0.5157 | 0.0159 | 0.1561 |  | 0.5016 | 0.0958 | 0.9283 |
|  |  | Significant |  |  | no | no | no | no | no | no | no | no | no |  | no | no | no |
| Δ*waaD* | 0.02% arabinose | dt (h) | 1.69 | 1.66 | 1.74 | 1.61 | 1.73 | 1.73 | 1.72 | 1.63 | 1.72 | 1.65 | 1.76 | 1.50 | 1.59 | 1.59 | 1.63 |
|  |  | dt (SD) | 0.03 | 0.02 | 0.04 | 0.06 | 0.01 | 0.04 | 0.03 | 0.05 | 0.08 | 0.05 | 0.03 | 0.07 | 0.03 | 0.07 | 0.04 |
|  |  | *t*-test | 0.1336 | - | 0.0361 | 0.2839 | 0.0047 | 0.0514 | 0.0449 | 0.3816 | 0.234 | 0.8022 | 0.0041 | 0.0146 | 0.0462 | 0.187 | 0.3726 |
|  |  | Significant | no |  | no | no | no | no | no | no | no | no | no | no | no | no | no |

^1^statistical analyses performed on growth curves for *E. coli* K-12 wild-type and Δ*waaD* conditional strains complemented with vector encoding *lptD* loop mutants shown in **Figures 3C**, **3D**, and **S3B**.

^2^dt(h) is the doubling time in hours calculated during log phase growth for biological triplicates.

^3^dt(SD) is the standard deviation for the doubling time during log phase growth of biological triplicates.

^4^an unpaired Student's t test was run using Prism 6.0 (GraphPad) Software and the Bonferroni correction was applied.

^5^significance was scored based on the unpaired Student's t-test.
