# Supplementary material for: Massive antibody discovery used to probe structure–function relationships of the essential outer membrane protein LptD
Source: eLife. 2019 Jun 25;8:e46258. doi: 10.7554/eLife.46258 (PMC6592684; doi:10.7554/eLife.46258)
Supplement: Supplementary file 2. — Names, descriptions, and references for all key resources (bacterial strains, plasmid constructs, and primers) as described in the text. [file elife-46258-supp2.docx]

**Storek et al. Supplementary File 2**

**Table. Strains, plasmids, and primers used in this study.**

| **IDENTIFIER** | **DESCRIPTION** |  |  |
| --- | --- | --- | --- |
|  |  |  |  |
| **Strain number** | **Strain name** | **Antibiotic resistance** | **Source** |
| GNE 115 | BW25113 |  |  |
| GNE 49 | BW25113 Δ*waaD::Kan* | Kan-R | Keio collection |
| GNE 92 | Δ*lptD*::P_BAD_-*lptD* | Carb-R, Kan-R | in-house |
| GNE 119 | Δ*lptD*::P_BAD_-*lptD, ΔwaaD::Cm* | Carb-R, Kan-R, Cm-R | in-house |
|  |  |  |  |
| **Plasmid** | **Description** | **Antibiotic resistance** | **Source** |
| pLMG18gm | pLMG18, IPTG-inducible, Cm-R swapped for Gent-R | Gent-R | in-house |
| pLDR9 | *attB* integration vector | Kan-R, Amp-R | ATCC |
| pLDR8 | temperature-sensitive lambda integrase plasmid | Kan-R | ATCC |
| pKD4 | lambda-red recombination | Kan-R | Addgene |
| pSIM18 | Expresses Lambda Red recombinase | Hygro-R | Chan et al. 2007 |
| pCP20 | temperature-sensitive FLP expression plasmid | Carb-R, Cm-R | Coli Genetic |
|  |  |  | Stock Center |
| pACYC184 | pACYC184 low copy plasmid | Tet-R, Cm-R | NEB |
| p*lptDWT* | pLMG18gm *lptD* | Gent-R | in-house |
| plptD*ΔL1* | pLMG18gm *lptD Δ237-239* | Gent-R | in-house |
| plptD*ΔL2* | pLMG18gm *lptD Δ264-269* | Gent-R | in-house |
| plptD*ΔL3* | pLMG18gm *lptD Δ293-309* | Gent-R | in-house |
| plptD*ΔL4* | pLMG18gm *lptD Δ335-352* | Gent-R | in-house |
| plptD*ΔL5* | pLMG18gm *lptD Δ382-387* | Gent-R | in-house |
| plptD*ΔL6* | pLMG18gm *lptD Δ420-425* | Gent-R | in-house |
| plptD*ΔL7* | pLMG18gm *lptD Δ462-476* | Gent-R | in-house |
| plptD*ΔL8* | pLMG18gm *lptD Δ532-546* | Gent-R | in-house |
| plptD*ΔL9* | pLMG18gm *lptD Δ588-601* | Gent-R | in-house |
| plptD*ΔL10* | pLMG18gm *lptD Δ629-634* | Gent-R | in-house |
| plptD*ΔL11* | pLMG18gm *lptD Δ661-680* | Gent-R | in-house |
| plptD*ΔL12* | pLMG18gm *lptD Δ706-711* | Gent-R | in-house |
| plptD*ΔL13* | pLMG18gm *lptD Δ740-745* | Gent-R | in-house |
|  |  |  |  |
|  |  |  |  |
|  |  |  |  |
| **Primers** | **Sequence** |  |  |
| *knock-outs* | |  |  |
| waaD KO F | GTCTGAGATTGTCTCTGACTCCATAATTCGAAGGTTACAGTTATGATCATC | | |
|  | gtgtaggctggagctgcttc |  |  |
| waaD KO F | CCCAAGACGGGCCGATCACCAGTATTTTCATGCAGAGCTCTTATGCGTCGCG | | |
|  | catatgaatatcctccttagttcctattc |  |  |
|  |  |  |  |
| *ΔlptD::P_BAD_-lptD* | |  |  |
| lptD KO F | GACCGTTTGTCACGCGCAACGTTACCGATGATGGAACAATAAAATCAACGT | | |
|  | tgtgtaggctggagctgcttcg |  |  |
| lptD KO R | ATTTCAATTAACCGCACTGCGGATTACGTGGTAAATCAACAAATCACAAA | | |
|  | catatgaatatcctcctta |  |  |
| LptD NheI F | gcgcgctagcaggaggaattcaccATGAAAAAACGTATCCCCACT | |  |
| lptD HindIII R | cgcgaagcttTCACAAAGTGTTTTGATACGG |  |  |
| pBAD-araC |  |  |  |
| lptD SacI F | AAAAGAGCTcgatgcataatgtgcctgtc |  |  |
| pBAD-araC |  |  |  |
| lptD SacI R | AAAAGAGCTcgttcaccgacaaacaacag |  |  |
|  |  |  |  |
| *pLMG18gm* | |  |  |
| gent F | GCACCAATAACTGCCTTAAAAAAAttaggtggcggtacttgg | |  |
| gent R | CAGGAGCTAAGGAAGCTAAAatgttacgcagcagcaac |  |  |
| pLMG18 F | TTTAGCTTCCTTAGCTCCTG |  |  |
| pLMG18 R | TTTTTTTAAGGCAGTTATTGGTGC |  |  |
|  |  |  |  |
| *pLMG18gm lptD* | |  |  |
| lptD F | gaattgtgagcggataacaattGCTAGCAGGAGGAATTCACCATGAAGAAGCGCATTCCGACC | | |
| lptD R | gtatcaggctgaaaatcttctcAAGCTTTCACAGGGTATTTTGGTACGG | |  |
| pLMG18gm F | GGTGAATTCCTCCTGCTAGCaattgttatccgctcacaattc |  |  |
| pLMG18gm R | CCGTACCAAAATACCCTGTGAAAGCTTgagaagattttcagcctgatac | |  |
