# Supplementary material for: Massive antibody discovery used to probe structure–function relationships of the essential outer membrane protein LptD
Source: eLife. 2019 Jun 25;8:e46258. doi: 10.7554/eLife.46258 (PMC6592684; doi:10.7554/eLife.46258)
Supplement: Supplementary file 3. — LptD peptides used for immunizations (Figure 1) were designed based on sequence conservation, surface exposure and loop location. [file elife-46258-supp3.docx]

**Storek et al. Supplementary File 3**

**Table. Sequences of linear and cyclic peptides used for immunizations.**

| **Group Number** | **Peptide Number** | **Peptide sequence** | **Linear or cyclic** | **LptD location** | **conserved epitope** |
| --- | --- | --- | --- | --- | --- |
| 1 | 1 | KVSDPSYFNDFDNKYGSSTDGY | linear | Loop 4 | no |
|  | 2 | FQVFSEQNTSSY | linear | Loop 5 | no |
|  | 3 | NTRDDMP | linear | Loop 6 | no |
|  | 4 | QTNLDWYNSRNTTKLDE | linear | Loop 7 | no |
|  | 5 | ASPEYIQATLPKYYSTAEQYKNGI | linear | Loop 11 | no |
|  | 6 | NGWDNDKQHAV | linear | Loop 13 | no |
| 2 | 7 | CKVSDPSYFNDFDNKYGSSTDGYC | cyclic | Loop 4 | no |
|  | 8 | CFQVFSEQNTSSYC | cyclic | Loop 5 | no |
|  | 9 | CNTRDDMPC | cyclic | Loop 6 | no |
|  | 10 | CQTNLDWYNSRNTTKLDEC | cyclic | Loop 7 | no |
|  | 11 | CASPEYIQATLPKYYSTAEQYKNGIC | cyclic | Loop 11 | no |
|  | 12 | CNGWDNDKQHAVC | cyclic | Loop 13 | no |
| 3 | 13 | YFTESRTGDDNIT | linear | Loop 9 | yes |
|  | 14 | NITWENDDKTG | linear | Loop 9 | yes |
|  | 15 | PYRDQSDIYN | linear | Loop 8 | yes |
|  | 16 | LPSDKVYEDEHPNDDSSRR | linear | Loop 3 | yes |
|  | 17 | PSDKVYEDEHPN | linear | Loop 3 | yes |
| 4 | 18 | CYFTESRTGDDNITC | cyclic | Loop 9 | yes |
|  | 19 | CNITWENDDKTGC | cyclic | Loop 9 | yes |
|  | 20 | CPYRDQSDIYNC | cyclic | Loop 8 | yes |
|  | 21 | CLPSDKVYEDEHPNDDSSRRC | cyclic | Loop 3 | yes |
|  | 22 | CPSDKVYEDEHPNC | cyclic | Loop 3 | yes |
